# A rare case of arterio-venous malformation of the left lower limb

**DOI:** 10.11604/pamj.2024.48.73.43960

**Published:** 2024-06-27

**Authors:** Souvik Sarkar, Ulhas Jadhav

**Affiliations:** 1 Department of Respiratory Medicine, Datta Meghe Institute of Higher Education and Research, Wardha, Maharashtra, India

**Keywords:** Arterio-venous malformation, left lower limb, left leg

## Image in medicine

A 20-year-old male presented to the outpatient department with swelling over the left lower limb from the past 5 years. The swelling was insidious in onset and gradually progressed over 5 years. It was painless initially but now had a dull aching pain which aggravated on walking and got relieved on taking rest. Upon examination, he had a normal build and was vitally stable. The swelling was present on the left lower limb over the shin, single in number with multiple lobules, of size 20 cm x 10 cm, ill-defined margins, with the normal overlying skin, no redness or signs of inflammation, no pulsations and no tenderness on touching. A bruit was heard over the swelling on auscultation with a stethoscope, raising suspicion of arterio-venous malformation. He was then subjected to a color doppler of the lower limb, which was suggestive of arterio-venous malformation (AVM). A left lower limb angiography was then done, which confirmed an AVM in the anterior and anterior-medial aspect of the knee, lower thigh, and upper leg with multiple feeding vessels from a saphenofemoral vein, popliteal artery, anterior tibial artery, and posterior tibial artery. An opinion of an interventional radiologist was then taken who advised to perform a left lower limb angioembolisation.

**Figure 1:**
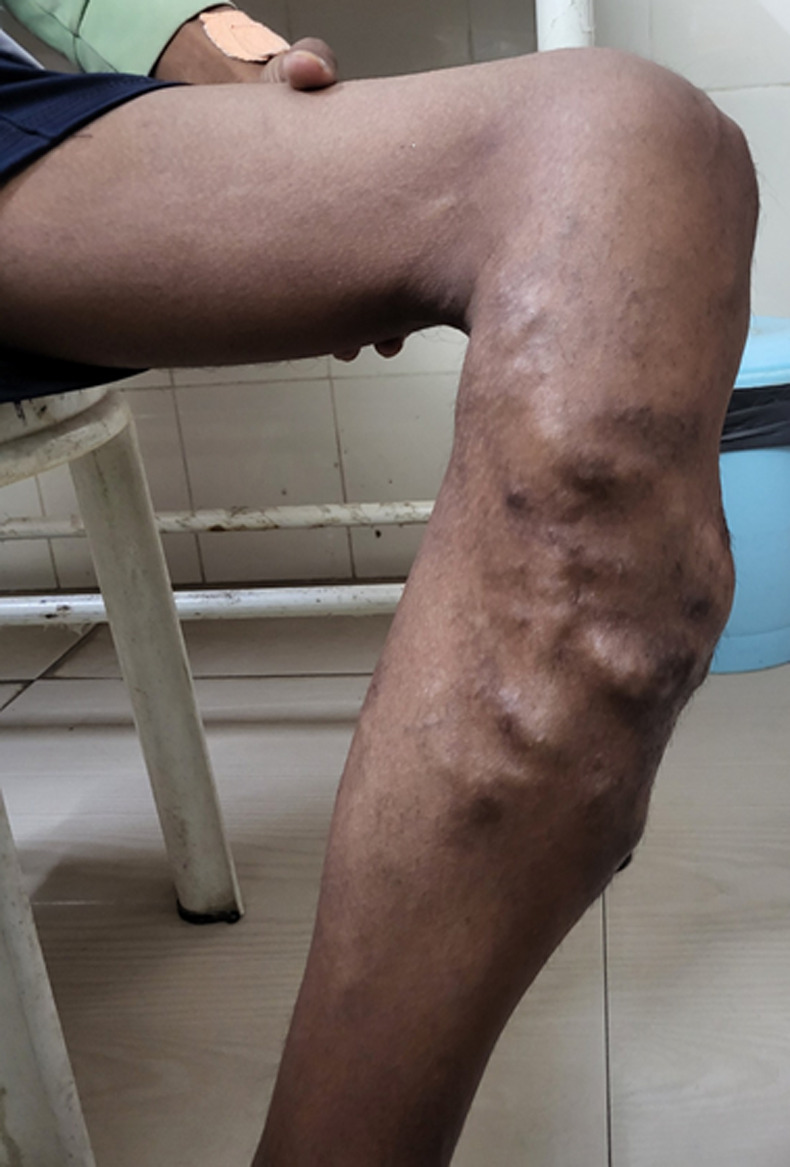
an ill-defined, multilobulated swelling of size 20 x 10 cm over the anterior-medial aspect of the left lower limb, suggestive of arterio-venous malformation

